# Can Men Have Milk Leg?

**Published:** 1892-01

**Authors:** Grace Danforth

**Affiliations:** Chicago Women’s Mediical College, Chicago


### Correspondence.

### Can Men Have Milk-Leg?

LETTER FROM DR. GRACE DANFORTH, CHICAGO WOMEN'S MEDICAL COLLEGE.

*Editor Daniel's Texas Medical Journal:*

Your JOURNAL is a regular visitor to the reading-room of the Woman's Medical College of Chicago, and is gleaned over more or less eagerly by all the students—by none with more interest than myself, and the president of the graduating class, Mrs. Herb of Houston, Texas. To-day the December issue is at hand; and the tortures of "la grippe" have been assuaged by merriment over Dr. Sears facing the possibility of ending his days as a woman! In this part of the world a man is not reduced to the dire extremity of a change of sex, to indulge in the luxury of *phlegmasia dolens*. Like hysteria, it is no longer supposed to be the exclusive monopoly of womankind. Recently a case of *phlegmasia dolens* in a man, was shown in one of the hospital clinics, following typhoid fever, and though thrombosis was the immediate cause, the general condition creating a phlebitis, was the same as in its more ordinary manifestation. It even favors the left leg in men, just the same as in women.

In the proceedings of the New York Academy of Medicine reported in a December issue of the *New York Journal of Medicine*, two of the doctors confessed having suffered with it, both following typhoid fever. Though the ætiology may be the same, if it will save the dignity of manhood, we can still continue to call it "milk leg" in a woman and "thombosis" in a man.

Advocates of the germ theory of disease are largely in the majority; though Dr. Sears would not stand entirely alone, even here, in his skepticism on the subject. In a lecture on "delusions in medicine," the speaker said that, "whereas in the past, people were satisfied to be delivered over to the worms after death, now science teaches that even during life we are perambulating colonies of worms." In its iconoclastic march, science had no respect for the sacred institutions of the past. A woman's mouth was considered as colonized by at least twenty different varieties of microbes; and when a lover bent over her with every fiber of his being thrilling in expectation of "sipping nectar fit for the gods," science would raise its profane hand, and shout "rats!" The gentlemen present were unanimously of the opinion that the time honored custom would not suffer at their hands, in spite of the warnings of science, and I quietly thought if women could stand the accompanying tobacco and whiskey, men might be daring enough to brave even twenty varieties of microbes.

I have wanted to extend the right hand of fellowship to the JOURNAL evei since its brave stand on the temperance question, but medical studies have claimed every instant of my time this fall. A short vacation for the holidays, and work will begin again with renewed interest, for it is in many ways a labor of love.

---

**The Value of Hygiene to Women.**—Dr. Schofield read a paper on this subject, and at the close of it proposed the following resolution, which was carried unanimously, viz.: "That this Congress hereby warmly advocate the instruction of girls and women in personal and domestic hygiene, as an integral part of their education."—*N, Y. Med. Record.*

